# RND transporters protect *Corynebacterium glutamicum* from antibiotics by assembling the outer membrane

**DOI:** 10.1002/mbo3.182

**Published:** 2014-06-18

**Authors:** Liang Yang, Shuo Lu, Juan Belardinelli, Emilie Huc-Claustre, Victoria Jones, Mary Jackson, Helen I Zgurskaya

**Affiliations:** 1 Department of Chemistry and Biochemistry, University of Oklahoma101 Stephenson Parkway, Norman, Oklahoma, 73019; 2 Department of Microbiology, Immunology and Pathology, Mycobacteria Research Laboratories, Colorado State UniversityFort Collins, Colorado, 80523-1619

**Keywords:** Cell envelope, membrane transport, resistance–nodulation–division family

## Abstract

*Corynebacterium–Mycobacterium–Nocardia* (CMN) group are the causative agents of a broad spectrum of diseases in humans. A distinctive feature of these Gram-positive bacteria is the presence of an outer membrane of unique structure and composition. Recently, resistance–nodulation–division (RND) transporters (nicknamed MmpLs, Mycobacterial membrane protein Large) have emerged as major contributors to the biogenesis of the outer membranes in mycobacteria and as promising drug targets. In this study, we investigated the role of RND transporters in the physiology of *Corynebacterium glutamicum* and analyzed properties of these proteins. Our results show that in contrast to Gram-negative species, in which RND transporters actively extrude antibiotics from cells, in *C. glutamicum* and relatives these transporters protect cells from antibiotics by playing essential roles in the biogenesis of the low-permeability barrier of the outer membrane. Conditional *C. glutamicum* mutants lacking RND proteins and with the controlled expression of either NCgl2769 (CmpL1) or NCgl0228 (CmpL4) are hypersusceptible to multiple antibiotics, have growth deficiencies in minimal medium and accumulate intracellularly trehalose monocorynomycolates, free corynomycolates, and the previously uncharacterized corynomycolate-containing lipid. Our results also suggest that similar to other RND transporters, Corynebacterial membrane proteins Large (CmpLs) functions are dependent on a proton-motive force.

## Introduction

Bacteria belonging to the *Corynebacterium–Mycobacterium–Nocardia* (CMN) group are the causative agents of a broad epidemiological, clinical, and pathological spectrum of diseases in humans and animals (Ventura et al. 2007). *Mycobacterium tuberculosis* and related species such as *Mycobacterium bovis* cause tuberculosis. *Nocardia* spp. are filamentous soil saprophytes, but also include pathogenic species that cause nocardiosis in humans and animals in the lung, central nervous system, brain, and skin. Historically *Corynebacterium* species are associated with diphtheroid bacillus, *Corynebacterium diphtheria*, a strictly human-adapted species, and the causative agent of diphtheria (Hadfield et al. 2000). *Corynebacterium jeikeium* is part of the normal human skin flora, but also implicated in a variety of nosocomial infections, most frequently associated with immunocompromised patients, and is typically multiresistant to clinically relevant antibiotics, with the exception of glycopeptides (Funke et al. 1997).

A distinctive feature of CMN species is the presence of cell walls with unique chemical composition and structure. CMN cell walls are extremely efficient permeability barriers and thought to be important determinants of virulence and antibiotic resistance of these bacteria (Puech et al. 2001; Louw et al. 2009). They are formed by a thick *meso-*diaminopimelic acid-containing peptidoglycan covalently linked to arabinogalactan, which in turn is esterified by long-chain *α*-alkyl, *β*-hydroxy fatty acids (mycolic acids). The mycolic acids form the inner leaflet of a pseudo-outer membrane where they interact with noncovalently associated (“free”) lipids including, in mycobacteria, trehalose dimycolate (TDM), and a variety of phospholipids and glycolipids (Brennan 2003). In corynebacteria, “free” lipids mainly include trehalose monocorynomycolate (TMCM), trehalose dicorynomycolate (TDCM), and some phospholipids (Bansal-Mutalik and Nikaido 2011). Mycolates apparently are major determinants of this permeability barrier because resistance to a variety of drugs is dependent on enzymes that are needed for mycolic acid synthesis and insertion into the envelope and no outer membrane is formed if mycolic acid biosynthesis is deficient (Puech et al. 2001; Zuber et al. 2008; Louw et al. 2009).

The synthesis and transport of CMN surface lipids requires transporters belonging to the resistance–nodulation–cell division (RND) superfamily, nicknamed MmpLs (Mycobacterial membrane protein Large) in mycobacteria (Jain et al. 2008; Grzegorzewicz et al. 2012; Varela et al. 2012). Mutants lacking these proteins are typically either defective in growth in vitro or significantly attenuated for virulence during host infection (Domenech et al. 2005). Just recently MmpL3 from *M. tuberculosis* (MmpL3tb) was identified as a target of at least eight new classes of antibiotics, of which SQ109, a 1,2-diamine compound related to ethambutol, entered clinical trials for the treatment of tuberculosis (Sacksteder et al. 2012; Tahlan et al. 2012). BLAST searches using MmpL3tb showed that the genomes of *M. tuberculosis* and *Nocardia farcinica* contain 13 and 9 *mmpL* homologs, respectively, whereas four MmpL homologs are found in the genome of *Corynebacterium glutamicum*. The number of MmpLs in genomes seems to correlate with the diversity of lipids and complexity of mycomembranes, as mycobacterial and corynebacterial cell walls are the most complex and the simplest among CMN, respectively.

By association with the RND superfamily, MmpLs are thought to be proton-motive force driven efflux transporters (Tseng et al. 1999). However, an experimental support for this notion is still missing. The archetypal RND efflux pump AcrB from *Escherichia coli* is a large protein comprising 12 transmembrane domains and two extracytoplasmic loops of ∼300 amino acids each (Murakami et al. 2002). This transporter and its close homologs from other Gram-negative bacteria comprise hydrophobe/amphiphile efflux-1 (HAE1) family within the RND superfamily (Tseng et al. 1999). HAE1 transporters function as drug:proton antiporters and expel from cells various antibiotics, detergents, and lipids (Nikaido 2011). Similar to AcrB, MmpLs are proposed to contain 12 TMDs, but these proteins do not share any sequence similarity with AcrB, vary significantly in their sizes (700–1200 kDa), and belong to a separate HAE2 family. For some of HAE2 proteins a direct involvement in efflux of antibiotics was proposed (Pasca et al. 2005). Others were assigned specific functions in lipid transport and biosynthesis (Jain et al. 2008). At least for one protein, MmpL7tb, the direct association with biosynthetic enzymes has been demonstrated using a yeast two-hybrid system (Jain and Cox 2005). For others, interaction with biosynthetic enzymes was proposed based on genetic analyses of biosynthetic pathways (Sondén et al. 2005; Seeliger et al. 2012; Wells et al. 2013).

In this study, we investigated the role of RND transporters (nicknamed CmpLs, Corynebacterial membrane proteins Large) in the physiology and cell envelope assembly of *C. glutamicum*. We found that mutants lacking these transporters are highly susceptible to multiple antibiotics. In agreement with previous studies (Varela et al. 2012), two CmpLs encoded in the genome of *C. glutamicum* CmpL1 (NCgl2769) and CmpL4 (NCgl0228) are essential for cell envelope biogenesis. Our results, however, show that these proteins function in two parallel reactions possibly involved in biosynthesis/transport of conjugated corynomycolates and phosphatidylinositol mannosides (PIMs) (Table1). We also found that growth defects in minimal medium associated with deletions of CmpLs are cumulative and worsen with progressive deletions of CmpL2 (NCgl0887) and CmpL3 (NCgl0599), suggesting that all four CmpLs contribute to the biogenesis of the *C. glutamicum* cell envelope.

**Table 1:** CmpLs of *Corynebacterium glutamicum*.

Name	Accession number for *C. glutamicum* ATCC13032	Size in a.a.	Closest homolog in *Mycobacterium tuberculosis* (identity, similarity)
CmpL1	Ncgl2769	772	MmpL3 (43%, 61%)
CmpL2	Ncgl0887	791	MmpL4 (31%, 47%)
CmpL3	Ncgl0599	730	MmpL8 (37%, 58%)
CmpL4	Ncgl0228	801	MmpL13 (28%, 47%)

CmpLs, Corynebacterial membrane proteins Large.

## Material and Methods

*C. glutamicum* and *Escherichia coli* strains and plasmids used in this study are listed in Table S1. Primers used in polymerase chain reaction (PCR) and construction of mutants are listed in Table S4.

### Culture conditions

*C. glutamicum* was grown at 30°C with aeration in brain–heart infusion (BHI) medium supplemented with 2% (w/v) glucose or 9.1% (w/v) sorbitol or in the minimal medium (20.9 g of 3-(N-Morpholino)propanesulfonic acid, 5 g of (NH_4_)_2_SO_4_, 5 g of urea, 1 g of citrate, 0.5 g of K_2_HPO_4_, 0.5 g of KH_2_PO_4_, 0.25 g of MgSO_4_·7H_2_O, 10 mg of CaCl_2_, 10 mg of MnSO_4_·H_2_O, 10 mg of FeSO_4_·7H_2_O, 10 mg of thiamine, 1 mg of ZnSO_4_·7H_2_O, 0.2 mg of CuSO_4_, 0.2 mg of biotin per liter, pH 7.3) with 1% glucose. *E. coli* DH5*α* and S17-1 were grown at 37°C in Luria-Bertani (LB) medium (10 g of Bacto-tryptone, 5 g of yeast extract, and 5 g of NaCl per liter). Kanamycin (25 *μ*g/mL), spectinomycin (100 *μ*g/mL for S17-1), or tetracycline (10 *μ*g/mL for DH5*α* and 20 *μ*g/mL for *C. glutamicum*) were added as needed.

### Construction of mutants

To construct knock-down and knock-in conditional mutants, the kanamycin resistance cassette of pAN6 (Frunzke et al. 2008) was replaced with tetracycline resistance Tc^R^ cassette. The Tc^R^ cassette was amplified by PCR using pDG1514 plasmid as a template and cloned into *Sma*I site of pAN6-based constructs. All constructs were verified by DNA sequencing (Oklahoma Medical Research Foundation).

In-frame deletion mutants of *C. glutamicum* ATCC13032 (WT) were constructed using a gene replacement approach (Schwarzer and Puhler 1991) with the help of the suicide vector pK19*mobsacB* (ATCC). The pK19mobsacB derivatives were transferred into *C. glutamicum* by conjugation with *E. coli* S17-1. The conjugation mixture was spread onto LB, Brain Heart Infusion, Sorbitol (LBHIS) agar plates (van der Rest et al. 1999) containing kanamycin and 50 *μ*g/mL of nalidixic acid (first recombination step) followed by the counter-screen on the LBHIS plates containing 10% (w/v) sucrose. The selected mutants were verified by PCR using primers listed in Table S4.

Conditional knock-down strains were constructed as described above, but the complementation plasmids pML1-Tc or pML4-Tc were electroporated into the clones of LY108 or LY109 subjected to the first recombination step with pK19mobsacB derivatives that generate in-frame deletions of *cmpL1* or *cmpL4* genes, respectively. The second crossover was next carried out on LBHIS plates containing 10% (w/v) sucrose and 0.1 mmol/L isopropyl-beta-D-thiogalactopyranoside (IPTG). Conditional knock-in strains carrying *lacI-P*_*tac*_*-cmpL1* or *lacI-P*_*tac*_*-cmpL4* in the intergenic region downstream of *ppc* gene were constructed by the same technique. But the *lacI-P*_*tac*_*-cmpL1* or *lacI-P*_*tac*_*-cmpL4* genes were inserted onto chromosomes of, respectively, LY109 and LY108 strains first, followed by in-frame deletions of *cmpL1* and *cmpL4* in their native locations.

### Drug susceptibility

Minimal inhibitory concentrations were determined using a serial twofold dilution approach (Tikhonova et al. 2002). Disk diffusion test was performed on BHI agar plates containing 4 × 10^6^ cells. The following antibiotics were tested: amikacin (50 *μ*g), ampicillin (250 *μ*g), carbenicillin (25 *μ*g), chloramphenicol (100 *μ*g), ethidium bromide (25 *μ*g), gentamicin (5 *μ*g), kanamycin (125 *μ*g), lincomycin (50 *μ*g), norfloxacin (50 *μ*g), novobiocin (50 *μ*g), rifampicin (50 *μ*g), spectinomycin (50 *μ*g), tobramycin (25 *μ*g), vancomycin (50 *μ*g). Plates were incubated at 30°C for 48 h. The antibiotic susceptibility was determined by measuring diameters of clearance zones.

### Lipid analyses

Noncovalently bound lipids were extracted from fresh or frozen-thawed cells using CMW (2:1:0.1) or reverse micelle solution (RMS) (10 mmol/L sulfosuccinic acid 1, 4-*bis*[2-ethylhexyl] ester sodium salt [AOT] in heptane) as described in Bansal-Mutalik and Nikaido (2011). Extracted lipids were separated by Thin Layer Chromatography (TLC) Silica gel 60 F_254_ (Merck KGaA, Darmstadt, Germany). Lipids from *C. glutamicum* WT and mutant strains were weighed and the same amount of each sample was loaded and run in C:M:W 30:8:1, 20:4:0.5, or 90:10:1. For 2D TLCs the lipids were developed in C:M:W (30:8:1) in the first direction and in hexane–diethyl ether–acetic acid (70:30:1) in the second direction.

Matrix-assisted laser desorption/ionization-Time-of-Flight mass spectrometry (MALDI-TOF) spectra were acquired in reflectron mode with a Bruker Ultraflex MALDI-TOF/TOF mass spectrometer (Bruker Daltonics, Billerica, MA). A total of 1000 shots were accumulated in positive ion mode, and mass spectrometry data were acquired with default calibration for the instrument. Lipids were loaded onto a metal plate with dihydroxybenzoic acid (5 mg/mL in CHCl_3_/CH_3_OH 1:1) as a matrix. For Liquid Chromatography-Mass Spectrometry lipids were resuspended in C:M:W (10:10:3) and run in both positive and negative mode using the method described by Sartain et al. (2011) on a high-resolution Agilent 6220 TOF mass spectrometer interfaced to a LC. For Gas Chromatography/Mass Spectrometry (GC/MS) analysis, the lipid samples were further treated with 3 mol/L HCl in CH_3_OH (Supelco, Bellefonte, PA) overnight at 80°C, dried and dissolved in 50 *μ*L of *N*,*O*-Bis(trimethylsilyl)trifluoroacetamide (Sigma, St. Louis, MO). Samples were heated at 60°C for 10 min prior to injection into the GC/MS. Analyses were carried out using a CP 3800 gas chromatograph (Varian, Agilent Technologies, Santa Clara, CA) equipped with an MS320 mass spectrometer in the electron impact mode and scanning from *m/z* 50 to *m/z* 1000 over 0.5 sec. Helium was used as the carrier gas with a flow rate of 1 mL min^−1^. The samples were run on a DB 5 column (10 m × 0.20 mm i.d.). The injector (splitless mode) was set for 335°C. The oven temperature was held at 50°C for 1 min, programed at 30°C min^−1^ to 130°C and then programed at 10°C min^−1^ to 330°C followed by a 10 min hold. The data analyses were carried out on a Varian WS data station.

### Protein analyses

For protein expression and purification, freshly transformed LY108(pML1), LY108(pML3), and LY109(pML4) were inoculated into 100 mL of 2x TY medium (16 g of Bacto-tryptone, 10 g of yeast extract, and 5 g of NaCl per liter) supplemented with 2% (w/v) glucose and 25 *μ*g/mL kanamycin and incubated at 30°C until OD_600_ reached ∼0.5. Protein expression was induced by 0.1 mmol/L IPTG for 8 h. Cells were collected by centrifugation at 4000*g* at 4°C for 20 min and resuspended in 50 mL buffer containing 10 mmol/L Tris-HCl, 1 mmol/L MgCl_2_, 1 mmol/L Phenylmethylsulfonyl fluoride (PMSF), and 100 *μ*g/mL DNase I (pH 8.0). Cells were broken by three or four rounds on French Press FA078 apparatus. Unbroken cells were removed by low-speed centrifugation at 4000*g* at 4°C for 10 min. Total membrane fractions were isolated by ultracentrifugation at 40,000*g* for 1 h at 4°C. Membrane proteins were solubilized in 10 mL of 100 mmol/L Tris-HCl (pH 8.0), 150 mmol/L NaCl, 1 mmol/L Ethylenediamine tetraacetic acid, 1 mmol/L PMSF, 1% *n*-dodecyl *β*-d-malto-pyranoside (DDM) for 3 h on ice. After removal of the insoluble fraction by ultracentrifugation, solubilized proteins were loaded onto a 0.4-mL column packed with Strep-tactin Superflow Agarose resin (Novagen, Merck KGaA). CmpL1 protein was purified from soluble fractions using the manufacturer's protocol modified to include 0.05% DDM in all buffers.

## Results

### Optimal growth of *C. glutamicum* requires functional RND proteins

**Figure 1:**
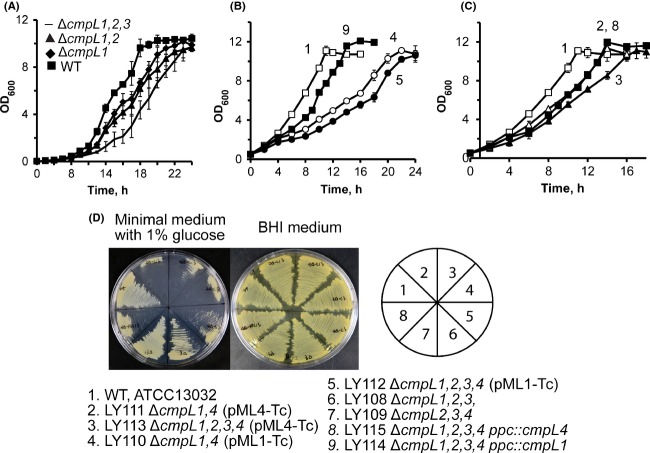
Growth and morphology of *Corynebacterium glutamicum* mutants lacking various combinations of *cmpL* genes. (A–C) Indicated strains were grown in minimal medium in the absence of IPTG with aeration at 30°C. Numbering of strains in (B–C) is the same as shown in (D). Doubling times for these and other mutant strains are shown in Table S2. (D) Indicated mutant strains were plated onto agar plates with the minimal (1% glucose) and BHI media. The cell growth was recorded after incubation for 48 h at 30°C. BHI, brain–heart infusion.

To investigate the functions of CmpL1, CmpL2, CmpL3, and CmpL4 (Table1), we systematically deleted the four *cmpL* genes in *C. glutamicum* ATCC 13032. Using a gene replacement approach based on the conditionally lethal *sacB* deletion marker from *Bacillus subtilis*, we successfully constructed single mutants deficient in each of the *cmpL* genes, as well as double and triple knock-out combinations of these genes (Tables2 and S1). Among the constructed mutants, the Δ*cmpL1* series had extended lag phases in minimal medium that were especially pronounced in the triple knock-out LY108 (Δ*cmpL1,2,3*) cells (Fig.1A). No growth defects were found in the rich BHI medium.

**Table 2:** Doubling times of *Corynebacterium glutamicum* strains grown in minimal medium supplemented with 1% glucose.

		Doubling time (h)	
Strains	Genotype	−IPTG	+0.1 mmol/L IPTG
ATCC13032	Wild type	2.98 ± 0.16	3.03 ± 0.22
LY100	Δ*cmpL1*	2.78 ± 0.11	ND
LY101	Δ*cmpL2*	2.98 ± 0.09	ND
LY102	Δ*cmpL3*	2.97 ± 0.03	ND
LY103	Δ*cmpL4*	2.84 ± 0.05	ND
LY104	Δ*cmpL1*,*3*	2.84 ± 0.04	ND
LY105	Δ*cmpL2,3*	2.79 ± 0.08	ND
LY106	Δ*cmpL2,4*	2.82 ± 0.17	ND
LY107	Δ*cmpL3,4*	2.89 ± 0.19	ND
LY108	Δ*cmpL1,2,3*	3.09 ± 0.02	ND
LY109	Δ*cmpL2,3,4*	2.81 ± 0.10	ND
LY110	Δ*cmpL1,4 (*pML1-Tc)	6.25 ± 0.09	ND^1^
LY111	Δ*cmpL1,4* z(pML4-Tc)	3.90 ± 0.10	4.14 ± 0.24
LY112	Δ*cmpL1,2,3,4* (pML1-Tc)	7.15 ± 0.28	ND^1^
LY113	Δ*cmpL1,2,3,4* (pML4-Tc)	4.80 ± 0.36	4.66 ± 0.20
LY114	Δ*cmpL1,2,3,4 ppc*::*lacI-*P_tac_-*cmpL1*	3.69 ± 0.04	ND
LY115	Δ*cmpL1,2,3,4 ppc:*:*lacI-*P_tac_-*cmpL4*	3.81 ± 0.07	ND^1^
LY116	Δ*cmpL1,2,3,4* (pML4-Tgt-Tc)	4.43 ± 0.14	4.48 ± 0.06

ND, not determined.

1 Toxic to cells.

**Figure 2:**
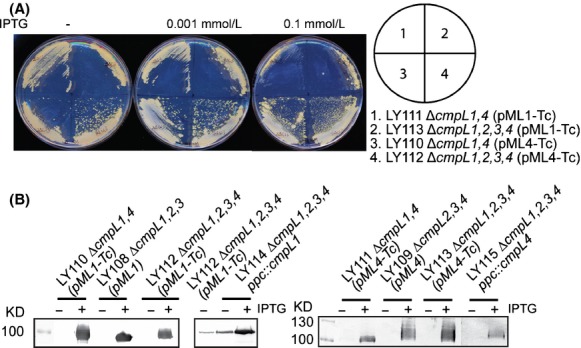
IPTG-dependent expression of CmpL1 and CmpL4 in conditional mutant strains of *Corynebacterium glutamicum*. (A) Conditional mutant strains were plated onto BHI agar supplemented with increasing concentrations of IPTG. Growth was recorded after incubation for 48 h at 30°C. (B) Immunoblotting analysis of membrane fractions isolated from indicated *C. glutamicum* strains incubated in the presence and absence of 0.1 mmol/L IPTG. Expression of CmpL1 and CmpL4 proteins was detected using a monoclonal anti-Strep-tag antibody.

Varela et al. (2012) previously reported a double Δ*cmpL1,4* mutant constructed using an insertional inactivation that has a severe growth defects in both minimal and the BHI media. However, despite multiple attempts neither deletion nor the insertional inactivation approaches yielded a double Δ*cmpL1,4* mutant. To establish the functions of CmpL1 and CmpL4, we constructed double LY110 and LY111 (Δ*cmpL1,4*) and, quadruple LY112 and LY113 (Δ*cmpL1,2,3,4*) mutants carrying plasmid-borne wild-type copies of *cmpL1* (pML1-Tc) or *cmpL4* (pML4-Tc) under control of the IPTG-inducible P_tac_ promoter. Although the second recombination event leading to the deletion of the chromosomal copies of *cmpL1* and *cmpL4* was carried out in the presence of IPTG, all final PCR-verified mutants were able to grow without IPTG suggesting that the P_tac_ promoter is leaky (Fig.1D). Indeed, immunoblotting with anti-Strep tag antibodies showed that CmpL1 was present in detectable amounts in the membrane fractions of *C. glutamicum* cells grown in the absence of IPTG but CmpL4 was produced below detection limits (Fig.2B). Both tagged proteins were overproduced in the presence of the inducer, with CmpL1 produced in quantities significantly higher than those of CmpL4.

The four conditional mutants grew notably slower than the WT strain in minimal medium but had no obvious growth defects in BHI medium (Fig.1B–D and Table2). A similar growth phenotype was reported in *C. glutamicum* mutants lacking mycoloyltransferases and defective in the outer lipid layer (Brand et al. 2003; Kacem et al. 2004). The growth defects were aggravated in the mutants lacking all four chromosomal copies of *cmpL* genes and were the most dramatic for LY110 and LY112 mutants that depend on *cmpL1* expression for growth (Fig.1B–C, Table2). Analysis of the chromosomal contexts showed that with the exception of *cmpL2*, the rest of *cmpL* genes are likely expressed in operons (Fig. S1). When compared to LY113, the coexpression of the plasmid-borne *cmpL4* and the downstream *tgt* gene in the quadruple LY116 mutant did not improve *C. glutamicum* growth in minimal medium (Fig.1A, Table2).

The presence of low concentrations of IPTG (0.001 mmol/L) partially alleviated the growth defects of the conditional mutants (Fig.2A). However, at higher concentrations, IPTG was detrimental for growth of cells carrying *P*_*tac*_*-cmpL1* on a plasmid (LY110, LY112) suggesting that overproduction of CmpL1 is toxic to the cells. To reduce toxicity of the plasmid-borne expression and increase the stringency of expression, *lacI-P*_*tac*_*-cmpL4* and *lacI-P*_*tac*_*-cmpL1* were inserted onto the chromosome in the ectopic location downstream of the PEP carboxylase *ppc* gene resulting in LY114 (Δ*cmpL1,2,3,4 ppc::lacI-P*_*tac*_*-cmpL4*) and LY115 (Δ*cmpL1,2,3,4 ppc::lacI-P*_*tac*_*-cmpL1*) strains (Fig. S1). The chromosomal expression did not increase the stringency of the promoter because both LY114 and LY115 mutants could grow in the absence of the inducer and CmpL1 was readily detected in uninduced LY115 cells by immunoblotting (Fig.2B). However, the toxic effects of the plasmid-borne expression were partially alleviated in the knock-in strains. LY115 cells grew better than LY110 and LY112 cells in minimal medium albeit slower (the doubling time 3.8 ± 0.1 h) than the wild-type strain (the doubling time 3.0 ± 0.2 h) (Fig.1A, Table2). This result further shows that deletions of *cmpL1* and *cmpL4* do not generate significant polar effects and that the phenotypes are at least partially complemented by plasmid-borne genes and genes in ectopic locations.

Taken together, these results show that all four CmpLs are required for *C. glutamicum* growth, albeit to a different degree. Although expression of *cmpL1* or *cmpL4* enables *C. glutamicum* growth in BHI, neither one of the two proteins is sufficient to fully complement growth defects in the minimal medium.

### Cells lacking both CmpL1 and CmpL4 are hypersusceptible to multiple antibiotics

We next investigated whether inactivation or overproduction of CmpLs affect the permeability properties of *C. glutamicum* cell envelope. For this purpose, we measured susceptibilities of various *cmpL* mutants to a broad range of antibiotics. We found that single, double, and triple *cmpL* mutants were indistinguishable from WT in their susceptibilities to antibiotics (Table S2). Thus, as long as at least one of CmpL1/CmpL4 is produced in the cells, as in the case of constructed double and triple deletion mutants, the cells are protected from antibiotics.

Inactivation of RND transporters in Gram-negative bacteria often does not produce any significant changes in antibiotic susceptibilities (Sulavik et al. 2001). However, overproduction of the same transporters leads to reduced susceptibility because of the increased efflux of antibiotics from cells (Nishino and Yamaguchi 2001). Similar observations have been reported with the MmpL7tb transporter, which when overproduced protected mycobacteria from antibiotics (Pasca et al. 2005). To determine whether overproduction of CmpL1, CmpL3, or CmpL4 could protect *C. glutamicum* against antibiotics, plasmids carrying the respective genes were introduced into LY108 (Δ*cmpL1,2,3*) or LY109 (Δ*cmpL2,3,4*) and susceptibility to antibiotics was measured using a disk diffusion assay. We found that in the absence of IPTG antibiotic susceptibilities of CmpL overproducers and WT were very similar (Table S3). In the presence of IPTG, LY108(pML1) cells overproducing CmpL1 became hypersusceptible to carbenicillin and novobiocin (Table S3), likely because of the toxicity of CmpL1 overproduction (Fig.2B). Thus, overproduction of either one CmpL1, CmpL 3, or CmpL4 does not protect cells from antibiotics.

**Figure 3:**
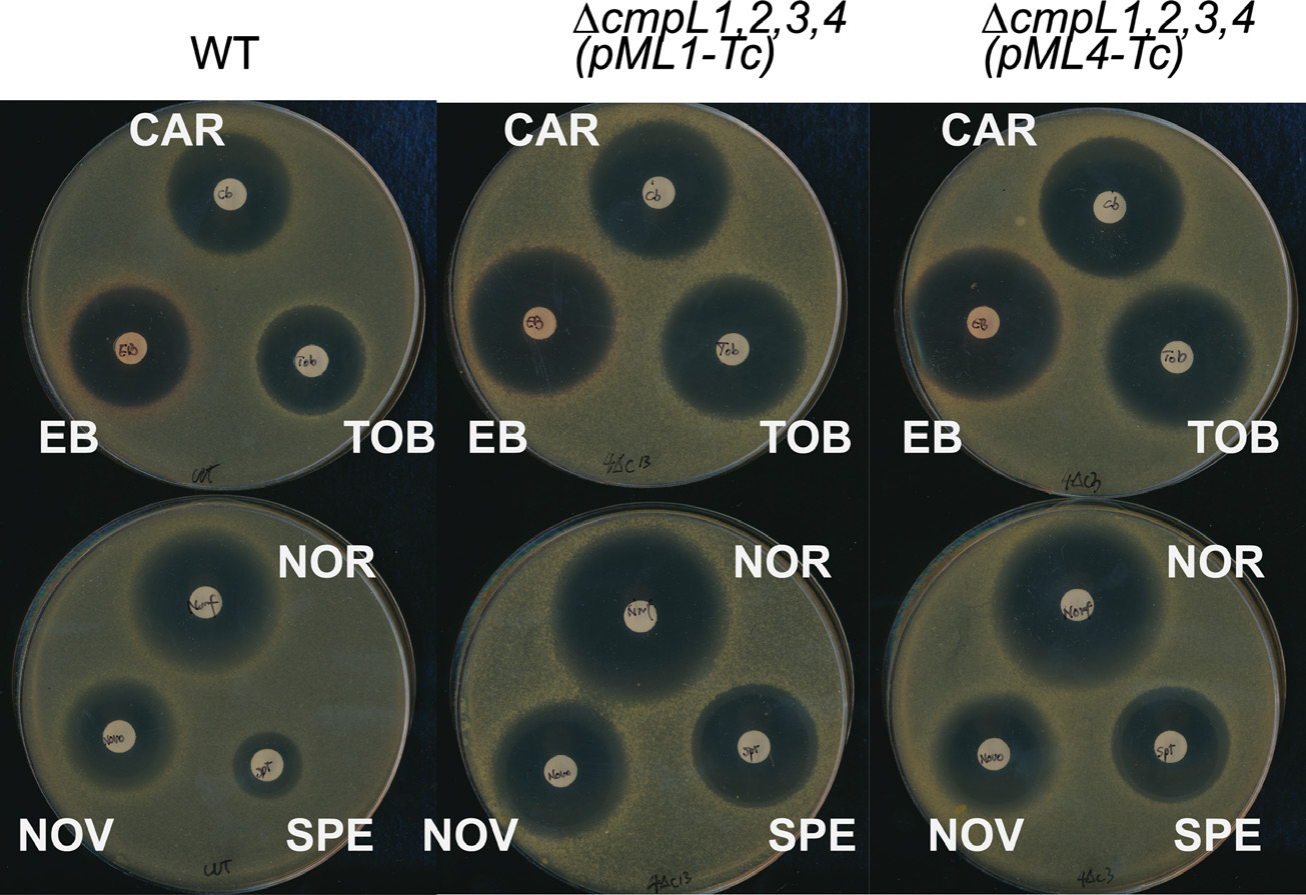
Antibiotic susceptibility of conditional mutant strains. BHI agar plates without IPTG were seeded with 4 × 10^6^ cells of indicated strains. Paper disks contained the following antibiotics: CAR, carbenicillin (25 *μ*g), EB, ethidium bromide (25 *μ*g), NOR, norfloxacin (50 *μ*g), NOV, novobiocin (50 *μ*g), SPE, spectinomycin (50 *μ*g), TOB, tobramycin (25 *μ*g). BHI, brain–heart infusion.

Susceptibility to various antibiotics was dramatically higher in the conditional mutants carrying deletions in all four chromosomal copies of *cmpLs*. Even in the rich BHI medium, which fully supports the growth, LY112 (Δ*cmpL1,2,3,4* [pML1-Tc]) and LY113 (Δ*cmpL1,2,3,4* [pML4-Tc]), conditional strains were hypersusceptible to carbenicillin, tobramycin, novobiocin, norfloxacin, spectinomycin, and ethidium bromide (Fig.3 and Table3). Interestingly, LY113 mutant with the modest growth defects in the minimal medium was somewhat more susceptible than LY112 to novobiocin, norfloxacin, spectinomycin, and tobramycin. The knock-in mutants LY114 (Δ*cmpL1,2,3,4 ppc::lacI-P*_*tac*_*-cmpL4*) and LY115 (Δ*cmpL1,2,3,4 ppc::lacI-P*_*tac*_*-cmpL1*) and LY116 (Δ*cmpL1,2,3,4* [pML4-Tgt-Tc]) coproducing CmpL4 along with the downstream Tgt protein displayed antibiotic susceptibility phenotypes intermediate between the WT and conditional mutant strains (Table3). Thus, the complementation of growth and cell envelope permeability defects depend on the copy number of *cmpL* genes. As in the growth assays, addition of IPTG did not lead to complementation of the drug hypersusceptibility phenotype and inhibited growth of CmpL1 overproducing strains at concentrations at or above 0.01 mmol/L.

**Table 3:** Antibiotic susceptibility of *Corynebacterium glutamicum* ATCC13032 and its mutants.

	Diameter of zones of clearance (mm)^1^					
Antibiotic	WT	LY112	LY113	LY116	LY114	LY115
Carbenicillin	25.9 ± 1.3	29.1 ± 1.3	29.8 ± 0.9	29.8 ± 1.4	26.8 ± 0.6	26.9 ± 1.0
Ethidium bromide	26.5 ± 2.0	31.2 ± 1.1	31.2 ± 1.2	30.8 ± 1.0	28.1 ± 0.6	28.7 ± 0.6
Norfloxacin	26.9 ± 0.9	30.3 ± 0.9	30.7 ± 1.4	30.3 ± 0.5	27.5 ± 0.5	28.0 ± 0.6
Novobiocin	20.0 ± 1.7	22.3 ± 1.5	23.2 ± 1.2	21.7 ± 0.9	21.0 ± 0.5	21.5 ± 0.5
Spectinomycin	13.2 ± 1.3	21.5 ± 1.5	22.2 ± 1.0	14.8 ± 1.30	13.4 ± 1.3	13.6 ± 1.2
Tobramycin	26.2 ± 2.5	30.6 ± 1.7	30.9 ± 1.6	29.4 ± 0.7	28.7 ± 0.6	28.6 ± 0.7

Numbers are averages of three independent experiments with standard deviations.

Taken together, these results suggest that *C. glutamicum* CmpLs are not involved in efflux of antibiotics. However, simultaneous depletion of both *cmpL1* and *cmpL4* genes makes cells hypersusceptible to multiple antibiotics, likely because of the changes in the composition or structure of *C. glutamicum* cell envelope.

### CmpLs function in the cell envelope biogenesis of *C. glutamicum*

Growth deficiency in minimal medium and increased susceptibility to antibiotics are indicative of defects in the biogenesis of *C. glutamicum* cell envelope. Analyses of *C. glutamicum* membranes purified from cells lacking and overproducing CmpL1 or CmpL4 protein in the absence of other *cmpLs* did not identify significant differences in their protein composition (Fig. S2). Hence, neither depletion nor significant overproduction of CmpLs affected the protein content of *C. glutamicum* cell membranes.

**Figure 4:**
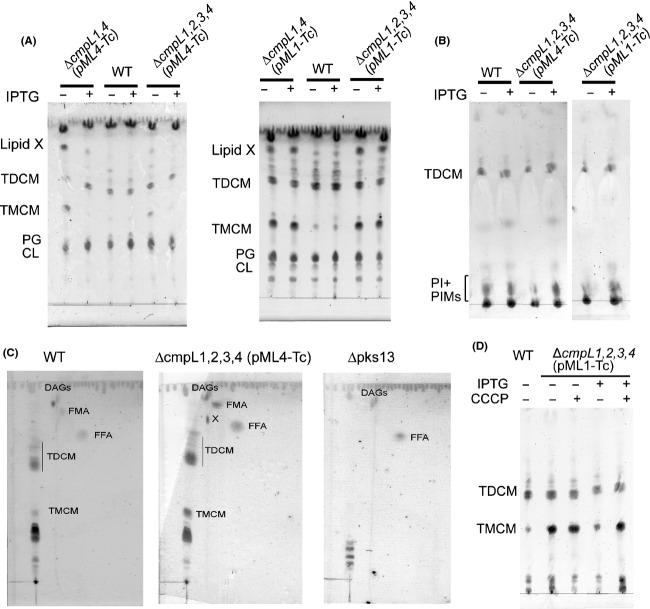
Composition of extractable lipids in *Corynebacterium glutamicum* mutant strains. (A) WT and mutant strains with the IPTG-controlled expression of CmpL1 or CmpL4 were grown in the minimal medium in the presence and absence of IPTG. Noncovalently associated lipids were extracted with CMW mixture and TLC was carried out with C:M:W 30:8:1 as the mobile phase. Lipids were stained with 5% phosphomolybdic solution. (B) Indicated strains were grown as in (A) but lipids were extracted with RMS mixture. After TLC separation in C:M:W 30:8:1, glycolipids were stained with anthrone spray. (C) 2D TLC analysis of lipid extracts from WT, LY113, and Δpks13 cells grown in the absence of IPTG. Lipids were developed in C:M:W (30:8:1) in the first direction and in hexane–diethyl ether–acetic acid (70:30:1) in the second direction. The identity of spots was confirmed by comparison to corynomycolate deficient Δpks13 cells and mass spectrometry. DAGs, diacylglycerols; FMA, free corynomycolates; FFA, free fatty acids; X, corynomycolate-containing lipid X. (D) LY113 (Δ*cmpL1,2,3,4* [*pML4-Tc*]) were grown in minimal medium. Induced (0.1 mmol/L IPTG) and control (no IPTG) cells were transferred into a fresh medium and incubated in the presence and absence of 0.2 mmol/L CCCP for 3–5 h at 30°C. Lipids were extracted with CMW, separated by TLC in C:M:W 30:8:1 and stained with anthrone.

We next analyzed the composition of extractable lipids in *C. glutamicum* WT and all constructed *cmpL* mutants. We found that the lipid composition of deletion mutants as well as LY114 (Δ*cmpL1,2,3,4 ppc::lacI-P*_*tac*_*-cmpL4*) and LY115 (Δ*cmpL1,2,3,4 ppc::lacI-P*_*tac*_*-cmpL1*) was similar to that of the WT cells (Fig. S3 and data not shown). In contrast, the profiles of extractable lipids from LY110 [Δ*cmpL1,4*(pML1-Tc]), LY112 (Δ*cmpL1,4* [pML4-Tc]), LY111 (Δ*cmpL1,2,3,4* [pML1-Tc]), and LY113 (Δ*cmpL1,2,3,4* [pML4-Tc]) strains were notably different from WT with several lipid species present in increased amounts (Fig.4). These profiles were also very different from those reported by Varela et al. (2012) for Δ*cmpL1,4* and Δ*cmpL1,2,4* mutants. The lipid profiles of Δ*cmpL1,4* and Δ*cmpL1,2,4* knock-out mutants were completely devoid of corynomycolates and were identical to those of Δ*pks13* (ΔNcgl2773) cells lacking the critical polyketide synthase required for the condensation step in the synthesis of corynomycolic acids and encoded downstream of *cmpL1* (Portevin et al. 2004; Varela et al. 2012). Hence, the depletion of CmpL1 and CmpL4 proteins in conditional mutants and the full simultaneous inactivation of *cmpL1* and *cmpL4* generate different bottlenecks in the synthesis of lipids and the assembly of cell envelopes.

Lipids accumulated in the LY113 cell envelope were identified using mass spectrometry and by comparison with lipid extracts from *C. glutamicum* Δ*pks13* mutant (Portevin et al. 2004). In the absence of IPTG, *C. glutamicum* mutants deficient in all four *cmpLs* accumulated large amounts of TMCM, free fatty acids, and corynomycolates. In addition, *cmpL* mutants accumulated an unknown lipid, which is absent in *pks13* mutant cells (lipid X in Fig.4C). A preliminary analysis of lipid X by LC/MS showed the presence of a corynomycolate-like component with the major M-H ion at *m/z* 537.4886 as well as components yielding M-1 ions at *m/z* of 563.5048 and 565.5201 (Fig. S4). These molecular weights are compatible with *O*-acetylated corynomycolates of saturated C-32, mono unsaturated C-34, and saturated C-34 such as are commonly found in Corynebacteria. Treatment of the sample with methanolic HCl followed by trimethylsilation (TMS) yielded the corresponding *O*-TMS corynomycolic acid methyl esters as analyzed by GC/MS (M-15 ions at *m/z* 567.5, 593.6, and 595.6) (Fig. S5). Thus, these preliminary data are consistent with lipid X being *O*-acetylated corynomycolates. Further work is required to confirm the structure of lipid X and determine whether another component or components are present in the Lipid X TLC spot.

The compositions of the extractable lipids from LY110, LY111, LY112, and LY113 were very similar (Fig.4A), suggesting that changes in lipid composition require the simultaneous limitation of both *cmpL1* and *cmpL4* pathways, whereas deletions of c*mpL2* and c*mpL3* do not affect significantly amounts of major lipids. Although conditional mutants complemented with CmpL1 had a more severe growth defects in minimal medium than those with CmpL4 (Fig.1), the lipid profiles of all conditional mutants were similar in the absence of IPTG. Hence, changes in lipid composition of membranes correlate with growth defects in minimal medium but do not necessarily cause these growth defects.

No TMCM or free corynomycolates were detected among lipids extracted from *cmpL* mutants with RMS, which specifically isolates free lipids located in the outer layer of the corynebacterial cell envelope (Bansal-Mutalik and Nikaido 2011) (Fig.4B). This result suggests that all these lipids are accumulated in the cytoplasmic membrane or the periplasm of mutant cells. Similar accumulation of TMM and free mycolates was previously reported for *M. tuberculosis* cells, in which expression of MmpL3tb was repressed by either genetic manipulations or by small molecule inhibitors (Grzegorzewicz et al. 2012). Thus, the functions of MmpLs and CmpLs appear to be conserved among CMN species and are related to the biogenesis of CMN cell walls. However, RMS extracts of *cmpL-*deficient cells contained lower amounts of TDCM and polar glycolipids, which by comparison to previous studies (Bansal-Mutalik and Nikaido 2011) could be PIMs. This result suggests that several lipid synthesis/transport pathways could be affected by simultaneous disruption of *cmpL1* and *cmpL4* genes.

Upon addition of IPTG, the overproduction of CmpL4 restored the balance of lipids in membranes of conditional mutants (Fig.4A–B). CmpL4 reduced the amounts of TMCM, free corynomycolates and lipid X to the WT levels and concurrently increased the amounts of TDCM in the RMS extracts of LY113 cells. In contrast, overproduction of CmpL1 did not reduce TMCM and free corynomycolate accumulation but the amounts of PIMs in the outer membrane (OM) increased in the IPTG-induced LY112 cells. These results suggest that functions of CmpL1 and CmpL4 are nonredundant and each protein uniquely affects the lipid biosynthesis/transport in *C. glutamicum*. However, non-native levels or timing of protein expression could also contribute to observed phenotypes.

### Proton-motive force is required for biogenesis of *C. glutamicum* cell walls

RND transporters of HAE1 family function as proton:drug antiporters that require a proton-motive force for their activities. To investigate whether a proton-motive force is required for the CmpL4-dependent complementation of the lipid content defects, LY113 (Δ*cmpL1,2,3,4* [pML4-Tc]) cells were grown in minimal medium in the presence and absence of the inducer IPTG and treated with 0.4 mmol/L carbonyl cyanide *m*-chlorophenyl hydrazone (CCCP) to dissipate the gradient of ions across the membrane. Treatment with CCCP reversed the complementation of lipid defects by overproduced CmpL4 leading to accumulation of TMCM and loss of TDCM (Fig.4D). Dissipation of ion gradient did not change the relative amounts of other lipids. Thus, the delivery of TDCM to the OM in LY113 cells by CmpL4 requires a proton-motive force.

## Discussion

How the elaborate outer membranes of CMN species are assembled remains unknown. In recent years, MmpLs emerged as major contributors to this process with mycobacterial MmpLs implicated in delivery of the variety of lipids to cell surfaces. In particular, MmpL3tb is an essential protein required for the export of TMM to the mycobacterial surface (Grzegorzewicz et al. 2012). MmpL7tb and MmpL8tb play respective roles in phthiocerol dimycoserosate (PDIM) and sulfolipid export (Converse et al. 2003; Jain et al. 2008), MmpL11tb appears to transport monomeromycoloyl diacylglycerol (MMDAG) and mycolate ester wax to the bacterial surface, whereas MmpL4 and MmpL5 are required for the export of mycobactins (Wells et al. 2013). Our results show that CmpLs play similar roles in the biogenesis of the corynebacterial cell envelope. *Corynebacterium glutamicum* CmpL1 and CmpL4 have parallel independent functions, disruption of which results in significant growth defects and accumulation of TMCM and free corynomycolates in internal layers of the cell walls and depletion of TDCM from the outer layer.

The double knock-out Δ*cmpL1,4* mutants lack all corynomycolates, suggesting that CmpL1/4 might be involved in biosynthesis of these lipids, possibly through association with Pks13 (Varela et al. 2012). In contrast, the conditional mutants in this study with either CmpL1 or CmpL4 as a limiting protein are fully proficient in the corynomycolate biosynthesis but cannot effectively conjugate corynomycolates to TMCM and other acceptors. These results suggest that depletion of CmpL1/4 mainly impacts the reactions downstream of biosynthesis such as transport of conjugated corynomycolates and that the corynomycolate biosynthesis in the knock-out mutants could be inhibited by a negative feedback regulation due to the overaccumulation of free corynomycolates.

The conditional mutant strains could grow without IPTG but further complementation of growth defects, albeit only partial, could be seen upon addition of the inducer (Figs.1 and 2). Hence the amounts of proteins needed for cell growth are quite low and the expression of these proteins generates an “all-or-nothing” effect. Such properties are consistent with CmpL1 and CmpL4 functioning in physiologically significant but not rate-limiting reactions.

Phenotypic and compositional analyses further showed that CmpL1 and CmpL4 are not interchangeable and function in parallel pathways. We found that the growth and lipid composition phenotypes differed in the conditional mutants carrying either c*mpL1* or *cmpL4* (Figs.1 and 4). LY110 and LY112 mutants relying for growth on CmpL1 had a more severe fitness problem in minimal medium than LY111 and LY113 mutants producing CmpL4 (Fig.1). These differences in severity of growth deficiencies correlated with differences in complementation of lipid defects. Overproduction of CmpL4 but not CmpL1 decreased TMCM levels and free corynomycolates levels to the WT levels (Figs.2 and 4). Similarly, CmpL4 (Ncgl0228) but not CmpL1 (Ncgl2769) complemented the lipid defects of Δ*cmpL1,4* strain (Varela et al. 2012). On the other hand, the overproduction of either CmpL4 or CmpL1 restored the amounts of PIMs in the OM. These results suggest that CmpL1 and CmpL4 perform different functions in *C. glutamicum* cells and that the severity of combining the two deletions together is caused by the loss of synergistic activities of these two proteins. Interestingly, the composition of glycolipids in LY114 and LY115 strains carrying *cmpL1* and *cmpL4* genes in ectopic chromosomal locations, respectively, was similar to that in WT even in the absence of IPTG. Yet both these strains had growth defects in the minimal medium (Fig.1 and Table2) and were hypersusceptible to various antibiotics (Table3). This result further supports the conclusion that deletions of *cmpLs* affect multiple biosynthetic pathways and have a cumulative effect on biogenesis of *C. glutamicum* cell envelope.

Despite similarities in the chemical composition and structure, certain differences in the assembly of cell walls have been noted between coryne- and mycobacteria. In particular, (1) *Corynebacterium* spp. can survive without biosynthesis of mycolates and hence without the OM, whereas isoniazid, an inhibitor of mycolic acid synthesis, is lethal to *Mycobacterium spp*.; (2) corynomycolate biosynthesis requires a type-I fatty acid synthase (FAS) and a polyketide synthase Pks13, whereas mycobacteria requires both type-I and type-II FAS complexes, modifying enzymes and Pks13; (3) the transfer of mycoloyl residues onto sugars can occur outside the plasma membrane in corynebacteria, but in mycobacteria at least some of the TMM is made in cytoplasm; and (4) antigens 85 catalyze mycoloyl transfer on the periplasmic side of the plasma membrane (Portevin et al. 2004; Radmacher et al. 2005; Kaur et al. 2009; Jackson et al. 2013). Surprisingly, the phenotypes of knock-down *C. glutamicum cmpL1* and *cmpL4* mutants (Fig.4) and *M. smegmatis* and *M. tuberculosis mmpL3* mutants (Grzegorzewicz et al. 2012) are similar and lead to accumulation of TMCM and TMM in the inner layers of cell walls, respectively. In addition, free corynomycolates and lipid X also accumulate in membranes further supporting the idea that the transfer of mycolates onto TMCM and possibly other cell envelope acceptors is affected. We conclude that this step in the biosynthesis of TDCM and TDM and delivery of these lipids to cell surfaces is conserved among CMN species and that functions of RND proteins are required for the successful completion of this step. In mycobacteria flipping of TMM from the cytosolic side of the inner membrane into the periplasm could be important in the biosynthesis of TDM from TMM. However, in corynebacteria the synthesis of TMCM is thought to occur in the periplasm (Tropis et al. 2005). Hence, it is unlikely that the deficiency in inner membrane transport by CmpLs is responsible for the accumulation of TMCM and the depletion of TDCM. Instead, we propose that in both species and similar to the situation with Gram-negative RND transporters, CmpL and MmpL proteins are involved in the transport of TMCM and TMM or free mycolates from the outer leaflet of the inner membrane to the site of TDM synthesis in the periplasm or in the outer membrane.

## Supporting Information

Figure S1 (A) Genetic contexts of four *cmpL* genes encoded on *Corynebacterium glutamicum* chromosome. (B) Localization and orientation of *cmpL1* and *cmpL4* genes expressed under the IPTG-inducible Ptac promoter in LY115 and LY116. **Figure S2.** Protein composition of membranes isolated from LY112 (Δ*cmpl1*,*2,3,4* [pML1-Tc]) and LY113 (Δ*cmpl1*,*2,3,4* [pML4-Tc]) in the presence and absence of IPTG. Membrane fractions were isolated by ultracentrifugation and analyzed by 12% SDS-PAGE. Overproduced CmpL1 and CmpL4 can be seen in unboiled samples (marked with asterisk). **Figure S3.** Composition of lipids extracted from *Corynebacterium glutamicum* mutants. (A) Cells were grown in the minimal medium and noncovalently associated lipids extracted with CMW mixture. TLC was carried out with C:M:W 30:8:1 as the mobile phase and lipids were stained with 5% phosphomolybdic solution. (B) Mutant cells were grown in minimal medium. Lipids were extracted either with CMW (left panel) or RMS (right) mixtures, separated on TLC plates with C:M:W 30:8:1 as the mobile phase and stained with anthrone. (C). Glycolipid composition of LY114 and LY115 knock-in mutant strains. Lipids were extracted from cells grown in minimal medium with CMW and analyzed as in (B). **Figure S4.** (A) LC/MS chromatogram of Lipid X. Component I which barely stuck to the reversed phase LC column was not further analyzed and may or may not be part of the Lipid X TLC spot. Component III is like component II except it is based on a C-34 fully saturated corynomycolate. (B) The mass spectrum of component II consistent with acetylated C-32 fully saturated corynomycolate (*m/z* = 537.4886) whose structure is shown on the right) and monounsaturated C-34 corynomycolate (*m/z* = 563.5049). **Figure S5.** The structure of the methyl ester trimethylsilyl ether of C-32 corynomycolate as determined by GC/MS after formation of the methyl ester and trimethylsilylation of the alcohol. The *m/z* 207 ion (asterisk) is from column bleed. M-15 is 567.6. The original LC/MS analysis also had a complex early eluting peak containing ions at *m/z* 485.3854, 489.3359, and 521.3622 and the GC/MS had many components in addition to the corynomycolates. These possible other components have not been investigated. **Table S1.** Strains and plasmids used in this study. **Table S2.** Antibiotic susceptibility of *Corynebacterium glutamicum* cells lacking *cmpLs*. **Table S3.** Antibiotic susceptibility of *Corynebacterium glutamicum* mutant cells overproducing various CmpL proteins. **Table S4.** Primers used in this study.
