# Supplementary material for: RND transporters protect Corynebacterium glutamicum from antibiotics by assembling the outer membrane
Source: Microbiologyopen. 2014 Jun 18;3(4):484–96. doi: 10.1002/mbo3.182 (PMC4287177; doi:10.1002/mbo3.182)
Supplement: Figure S1 — (A) Genetic contexts of four cmpL genes encoded on Corynebacterium glutamicum chromosome. (B) Localization and orientation of cmpL1 and cmpL4 genes expressed under the IPTG-inducible Ptac promoter in LY115 and LY116. Figure S2. Protein composition of membranes isolated from LY112 (Δcmpl1,2,3,4 [pML1-Tc]) and LY113 (Δcmpl1,2,3,4 [pML4-Tc]) in the presence and absence of IPTG. Membrane fractions were isolated by ultracentrifugation and analyzed by 12% SDS-PAGE. Overproduced CmpL1 and CmpL4 can be seen in unboiled samples (marked with asterisk). Figure S3. Composition of lipids extracted from Corynebacterium glutamicum mutants. (A) Cells were grown in the minimal medium and noncovalently associated lipids extracted with CMW mixture. TLC was carried out with C:M:W 30:8:1 as the mobile phase and lipids were stained with 5% phosphomolybdic solution. (B) Mutant cells were grown in minimal medium. Lipids were extracted either with CMW (left panel) or RMS (right) mixtures, separated on TLC plates with C:M:W 30:8:1 as the mobile phase and stained with anthrone. (C). Glycolipid composition of LY114 and LY115 knock-in mutant strains. Lipids were extracted from cells grown in minimal medium with CMW and analyzed as in (B). Figure S4. (A) LC/MS chromatogram of Lipid X. Component I which barely stuck to the reversed phase LC column was not further analyzed and may or may not be part of the Lipid X TLC spot. Component III is like component II except it is based on a C-34 fully saturated corynomycolate. (B) The mass spectrum of component II consistent with acetylated C-32 fully saturated corynomycolate (m/z = 537.4886) whose structure is shown on the right) and monounsaturated C-34 corynomycolate (m/z = 563.5049). Figure S5. The structure of the methyl ester trimethylsilyl ether of C-32 corynomycolate as determined by GC/MS after formation of the methyl ester and trimethylsilylation of the alcohol. The m/z 207 ion (asterisk) is from column bleed. M-15 is 567.6. The original LC/MS an [file mbo30003-0484-sd1.pdf]

Supplemental Data

RND transporters protect *Corynebacterium glutamicum* from antibiotics by assembling the outer membrane

Liang Yang[a], Shuo Lu[a], Juan Belardinelli[b], Emilie Huc-Claustre[b], Victoria Jones[b], Mary Jackson[b], Helen I. Zgurskaya[a,1]

[a] University of Oklahoma, Department of Chemistry and Biochemistry, 101 Stephenson Parkway, Norman, OK 73019;

[b] Mycobacteria Research Laboratories; Department of Microbiology, Immunology and Pathology; Colorado State University

[1] To whom correspondence should be addressed. E.mail: elenaz@ou.edu

Table S1. Strains and plasmids used in this study

Strain/plasmid	Description	Source
E. coli		
DH5α	*supE44 ΔlacU169 hsdR17 recA1 gyrA96 thi-1 relA1*	
S17-1	*recA pro hsdR RP4-2*-Tc::Mu-Km::Tn7	
C. glutamicum		
WT	Wild type *C. glutamicum* ATCC13032	ATCC
Δpks13	ATCC13032 *Δpks13::km*	(Portevin *et al.*, 2004)
LY100	Δ*cmpL1* (*Ncgl2769*)	This study
LY101	Δ*cmpL2* (*Ncgl0887*)	This study
LY102	Δ*cmpL3* (*Ncgl0599*)	This study
LY103	Δ*cmpL4*(*Ncgl0228*)	This study
LY104	Δ*cmpL1* Δ*cmpL3*	This study
LY105	Δ*cmpL2* Δ*cmpL3*	This study
LY106	Δ*cmpL2* Δ*cmpL4*	This study
LY107	Δ*cmpL3* Δ*cmpL4*	This study
LY108	Δ*cmpL1* Δ*cmpL2* Δ*cmpL3*	This study
LY109	Δ*cmpL2* Δ*cmpL3* Δ*cmpL4*	This study
LY110	Tc^R; Δ*cmpL1* Δ*cmpL4 (*pML1-Tc)	This study
LY111	Tc^R; Δ*cmpL1* Δ*cmpL4 (*pML4-Tc)	This study
LY112	Tc^R; Δ*cmpL1* Δ*cmpL2* Δ*cmpL3* Δ*cmpL4* (pML1-Tc)	This study
LY113	Tc^R; Δ*cmpL1* Δ*cmpL2* Δ*cmpL3* Δ*cmpL4* (pML4-Tc)	This study
LY114	Δ*cmpL1* Δ*cmpL2* Δ*cmpL3* Δ*cmpL4 ppc*::*lacI*-P_{tac}-*cmpL1*	This study
LY115	Δ*cmpL1* Δ*cmpL2* Δ*cmpL3* Δ*cmpL4 ppc*::*lacI*-P_{tac}-*cmpL4*	This study
LY116	Tc^R; Δ*cmpL1* Δ*cmpL2* Δ*cmpL3* Δ*cmpL4* (pML4-Tgt-Tc)	This study
Plasmids		
pAN6	Kan^R; *C. glutamicum*/*E. coli* shuttle vector	(Frunzke *et al.*, 2008)
pK19*mob-sacB*	Kan^R; vector for allelic exchange in *C. glutamicum* (pK18*oriV*$_{E.c.}$*sacB lacZ*α)	(Schafer *et al.*, 1994)
pML1	Kan^R; pAN6 derivative containing P_{tac}-*cmpl1*	This study
pML3	Kan^R; pAN6 derivative containing P_{tac}-*cmpl3*	This study
pML4	Kan^R; pAN6 derivative containing P_{tac}-*cmpl4*	This study
pDG1514	Tc^R; plasmid contains a tetracycline cassette	(Guerout-Fleury *et al.*, 1995)
pML1-Tc	Tc^R; pML1 with tetracycline cassette	This study
pML4-Tc	Tc^R; pML4 with tetracycline cassette	This study
pML4-Tgt-Tc	Tc^R; pAN6 derivative containing P_{tac}-*cmpL4-tgt* (N*cgl0228-0229*)	This study

Table S2. Antibiotic susceptibility of *C. glutamicum* cells lacking *cmpLs*.

Strain	MIC (μg/ml)										
	CAR	CHL	RIF	LIN	NOR	VAN	NOV	GM	TOB	SPE	EB
WT	3-6	3	0.0125	6.25	1.5-3	0.38	6.25-12.5	0.19	0.1-0.2	31.25-62.5	3.125-6.25
LY100	3-6	3	0.0125	6.25	1.5-3	0.38	6.25-12.5	0.19	0.1-0.2	31.25-62.5	1.56-3.125
LY103	3-6	3	0.0125	6.25	1.5-3	0.38	6.25-12.5	0.19	0.1-0.2	31.25-62.5	1.56-3.125
LY104	3-6	3	0.0125	6.25	1.5-3	0.38	6.25-12.5	0.19	0.1-0.2	31.25-62.5	1.56-3.125
LY106	3-6	3	0.0125	6.25	1.5-3	0.38	6.25-12.5	0.19	0.1-0.2	31.25-62.5	1.56-3.125
LY107	3-6	3	0.0125	6.25	1.5-3	0.38	6.25-12.5	0.19	0.1-0.2	31.25-62.5	1.56-3.125
LY108	3-6	3	0.0125	6.25	1.5-3	0.38	6.25-12.5	0.19	0.1-0.2	31.25-62.5	1.56-3.125
LY109	3-6	3	0.0125	6.25	1.5-3	0.38	6.25-12.5	0.19	0.1-0.2	31.25-62.5	1.56-3.125

CAR, carbenicillin; CHL, chloramphenicol; RIF, rifampiicin; VAN, vancomycin;, LIN, lincomycin; NOR, norfloxacin; NOV, novobiocin; GM, gentamycin; TOB, tobramycin; SPE, spectinomycin; EB, ethidium bromide

Table S3. Antibiotic susceptibility of *C. glutamicum* mutant cells overproducing various CmpL proteins.

Strains	IPTG (mM)	Diameters (mm)					
		CAR	NOR	SPE	TOB	EB	NOV
WT(pAN6)	-	25.9±0.2	26.8±0.3	13.9±0.7	27.5±0.4	28.0±0.7	21.1±0.7
	0.1	25.8±0.3	27.3±0.3	13.8±0.3	28.5±0.5	28.5±0.5	21.8±0.3
LY108(pML1)	-	25.6±0.4	26.4±0.4	13.4±0.4	28.5±0.5	27.8±0.2	21.8±0.3
	0.01	27.6±1.1	26.9±1.1	13.4±0.4	28.4±0.4	27.9±0.2	22.7±0.9
LY108(pML3)	-	25.5±0.5	26.5±0.5	14.0±0.8	27.8±0.3	27.3±0.5	22.4±0.4
	0.01	25.8±0.7	26.8±0.6	13.4±0.4	28.3±0.5	27.7±0.7	22.3±0.6
LY109(pML4)	-	25.5±0.4	27.5±0.3	13.8±0.6	28.3±0.3	28.0±0.8	21.8±0.2
	0.1	25.9±0.7	27.7±0.5	14.1±1.1	30.0±0.8	27.7±0.5	22.3±0.6

Table S4 Primers used in this study

Name	Sequences(5’-3’)
Primers used for crossover PCR	
Cmpl1-del-1	ACTGAATTCTCCTTCTAGTGTGGGACGCTC
Cmpl1-del-2	CCCATCCACTAAACTTAAACAAGCTTGGTGCCGAAAATGAC
Cmpl1-del-3	TGTTTAAGTTTAGTGGATGGGCTCAACGACGATGAGGAAGTCACC[illegible]
Cmpl1-del-4	ATTGAATTCTTTTCGACGAAGCCCCACTCCC
Cmpl2-del-1	TACGAGGTCTGCGGTGGCAATG
Cmpl2-del-2	CCCATCCACTAAACTTAAACACCGCCAGCGTCGTGATACCTATGAG
Cmpl2-del-3	TGTTTAAGTTTAGTGGATGGGAAGGAAAATGTAGGTGTCGG
Cmpl2-del-4	ACTGAATTCAATGCGACTCCATCTTCTATG
Cmpl3-del-1	ACTGCATGCCCAATGAGAGCAGCGGCAATT
Cmpl3-del-2	CCCATCCACTAAACTTAAACAGGGCAGAAAGATTCTCATCAAGCG
Cmpl3-del-3	TGTTTAAGTTTAGTGGATGGGTGGCCGTCAAAATTGTCCAATCAG
Cmpl3-del-4	ACTTCTAGATCTCTAGCCGTTTTTTGC
Cmpl4-del-1	ACTGTCGACTCCCGTACTTGTTCAGGATTAG
Cmpl4-del-2	CCCATCCACTAAACTTAAACAGAATAGCAATTTCGCCACGG
Cmpl4-del-3	TGTTTAAGTTTAGTGGATGGGCGGGCGTGTTTTTCGATGCTTTC
Cmpl4-del-4	TTCGTCGACAAGCGCCATGC
Primers used to verify mutants	
Cmpl1-mut-f	CAATCCCACTGACAAAGAAGGC
Cmpl1-mut-r	CGGTGAGCGAGACCGAGC
Cmpl2-mut-f	CATGATTGCGCGTTGTCG
Cmpl2-mut-r	TTCTTAATCCAGGCGCATGC
Cmpl3-mut-f	GTTAATTTTCTGGAATGCC
Cmpl3-mut-r	TGAGTCGGCTAAAAACAG
Cmpl4-mut-f	GCTAACAAGGTTCTGGGTG
Cmpl4-mut-r	CAGATTTGAAATCCACGC
Primers used for construction of expression plasmids	
Cmpl1-Exp-f	TAATCCACATATGTTTTCTAAATGGGGCCACTTTG
Cmpl1-Exp-r	ATATTCGCGCTAGCATCTAGATCCTCAAGCCTG
Cmpl3-Exp-f	GGCGGCATATGTCTACTAGCATCACAACAGAG
Cmpl3-Exp-r	GGATGTCTAGATAGCTGAGGCTGCTTCTG
Cmpl4-Exp-f	GAAGAACCATATGGCGAAATTGCTATTCAGG
Cmpl4-Exp-r	TAATAATAGCTAGCACGTGCAGCCTGCTTCTC
Cmpl4+Tgt-Exp	TCCTGATA GCTAGCTGAAACCTTCGACGCGTAG
Primers for amplification of tetracycline cassette	
Tc cassette-f	CAAGCCCGGGAACATGAGAATTCCTG
Tc cassette-r	TTCGCCCGGGTCTGCAGATAGTGTAC

References

Frunzke, J., V. Engels, S. Hasenbein, C. Gatgens & M. Bott, (2008) Co-ordinated regulation of gluconate catabolism and glucose uptake in Corynebacterium glutamicum by two functionally equivalent transcriptional regulators, GntR1 and GntR2. *Mol Microbiol* 67: 305-322.

Guerout-Fleury, A.M., K. Shazand, N. Frandsen & P. Stragier, (1995) Antibiotic-resistance cassettes for Bacillus subtilis. *Gene* 167: 335-336.

Portevin, D., C. de Sousa-D'Auria, C. Houssin, C. Grimaldi, M. Chami, M. Daffé & C. Guilhot, (2004) A polyketide synthase catalyzes the last condensation step of mycolic acid biosynthesis in mycobacteria and related organisms. *Proceedings of the National Academy of Sciences* 101: 314-319.

Schafer, A., A. Tauch, W. Jager, J. Kalinowski, G. Thierbach & A. Puhler, (1994) Small mobilizable multi-purpose cloning vectors derived from the Escherichia coli plasmids pK18 and pK19: selection of defined deletions in the chromosome of Corynebacterium glutamicum. *Gene* 145: 69-73.

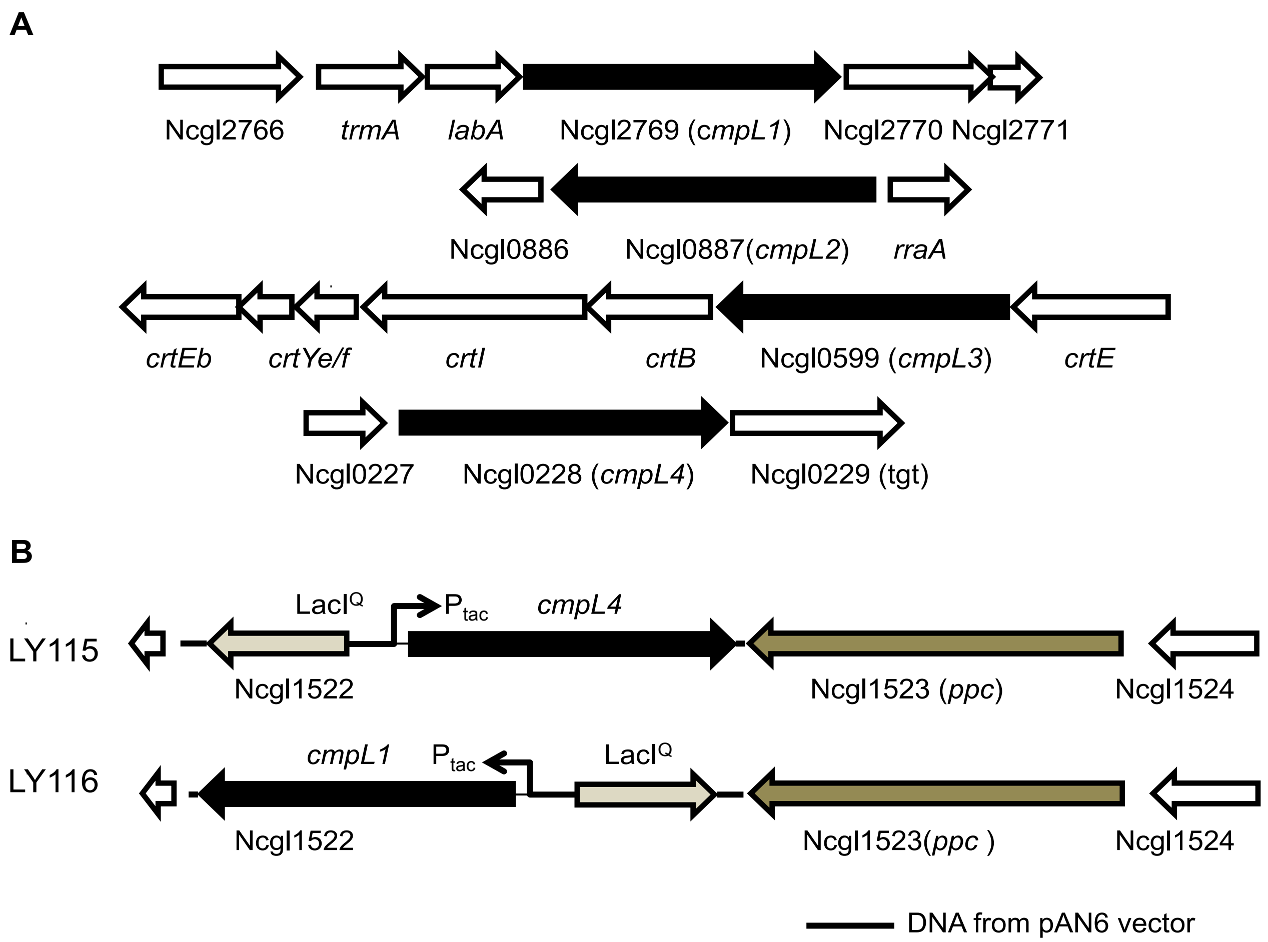

Fig. S1. (A) Genetic contexts of four *cmpL* genes encoded on *C. glutamicum* chromosome. (B) Localization and orientation of *cmpL1* and *cmpL4* genes expressed under the IPTG -inducible Ptac promoter in LY115 and LY116.

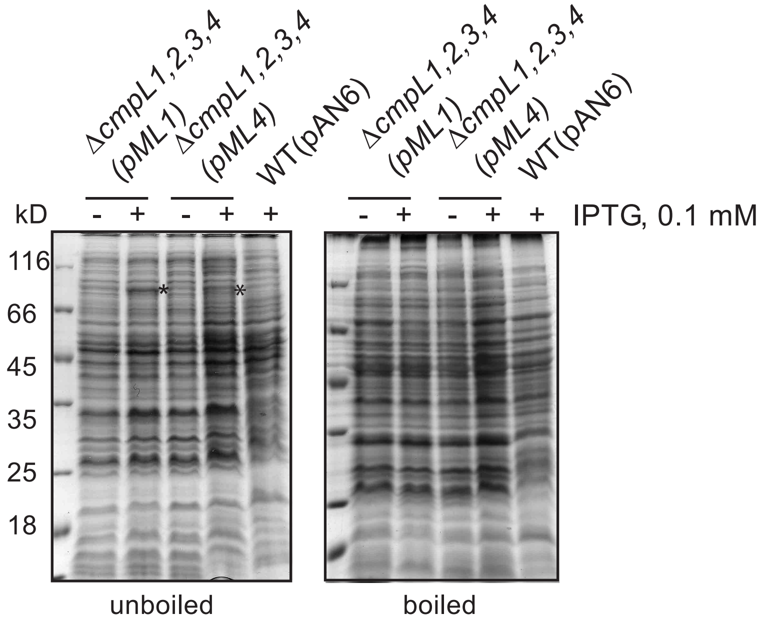

Fig. S2. Protein composition of membranes isolated from LY112 (Δ*cmpl1,2,3,4* (pML1-Tc)) and LY113 (Δ*cmpl1,2,3,4* (pML4-Tc)) in the presence and absence of IPTG. Membrane fractions were isolated by ultracentrifugation and analyzed by 12% SDS-PAGE. Overproduced CmpL1 and CmpL4 can be seen in unboiled samples (marked with asterisk).

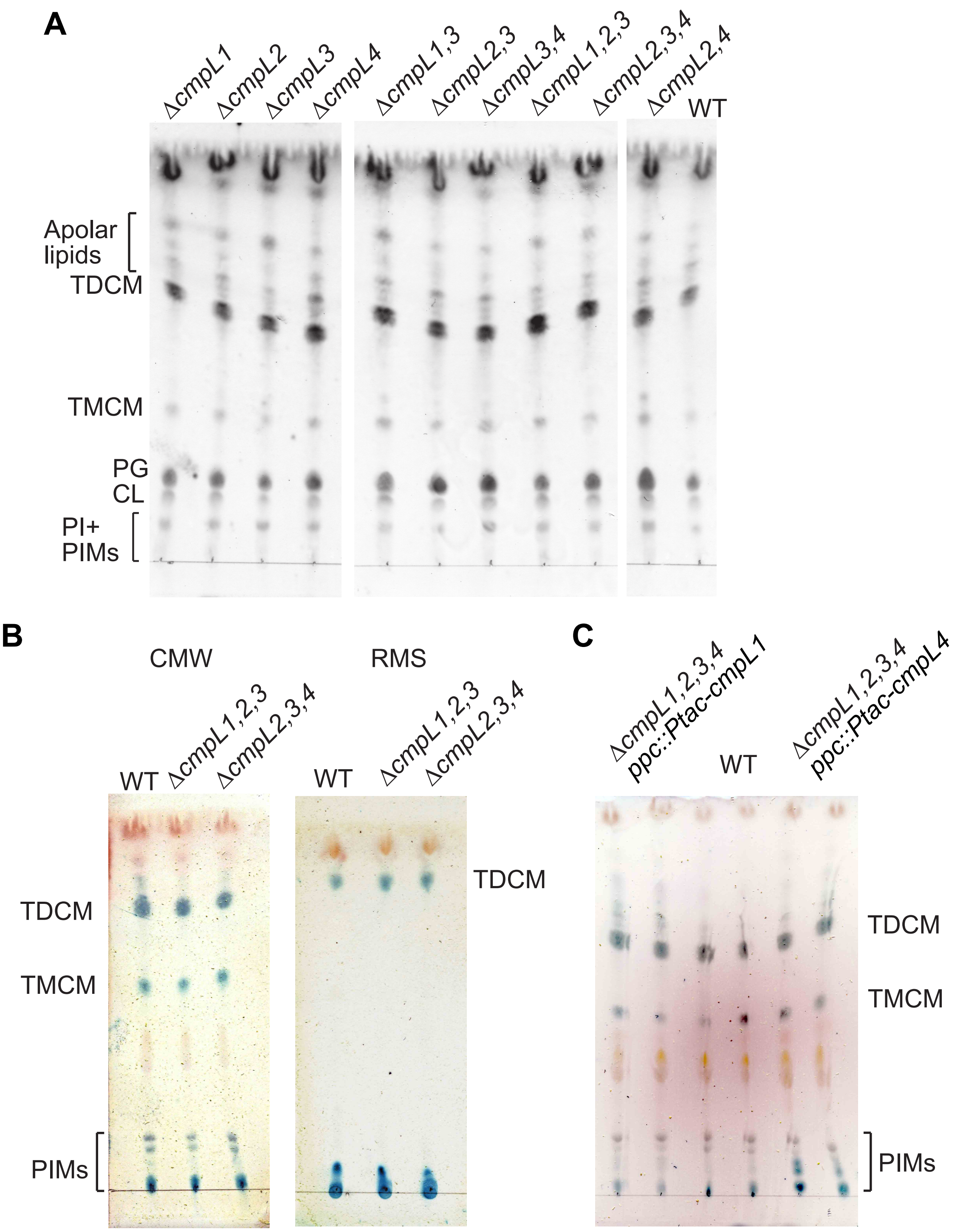

Fig. S3. Composition of lipids extracted from *C. glutamicum* mutants. (A) Cells were grown in the minimal medium and noncovalently associated lipids extracted with CMW mixture. TLC was carri out with C:M:W 30:8:1 as the mobile phase and lipids were stained with 5% phosphomolybdic solution. (B) Mutant cells were grown in minimal medium. Lipids were extracted either with CMW panel) or RMS (right) mixtures, separated on TLC plates with C:M:W 30:8:1 as the mobile phase stained with anthrone. (C). Glycolipid composition of LY114 and LY115 knockin mutant strains. Lipids were extracted from cells grown in minimal medium with CMW and analyzed as in (B).

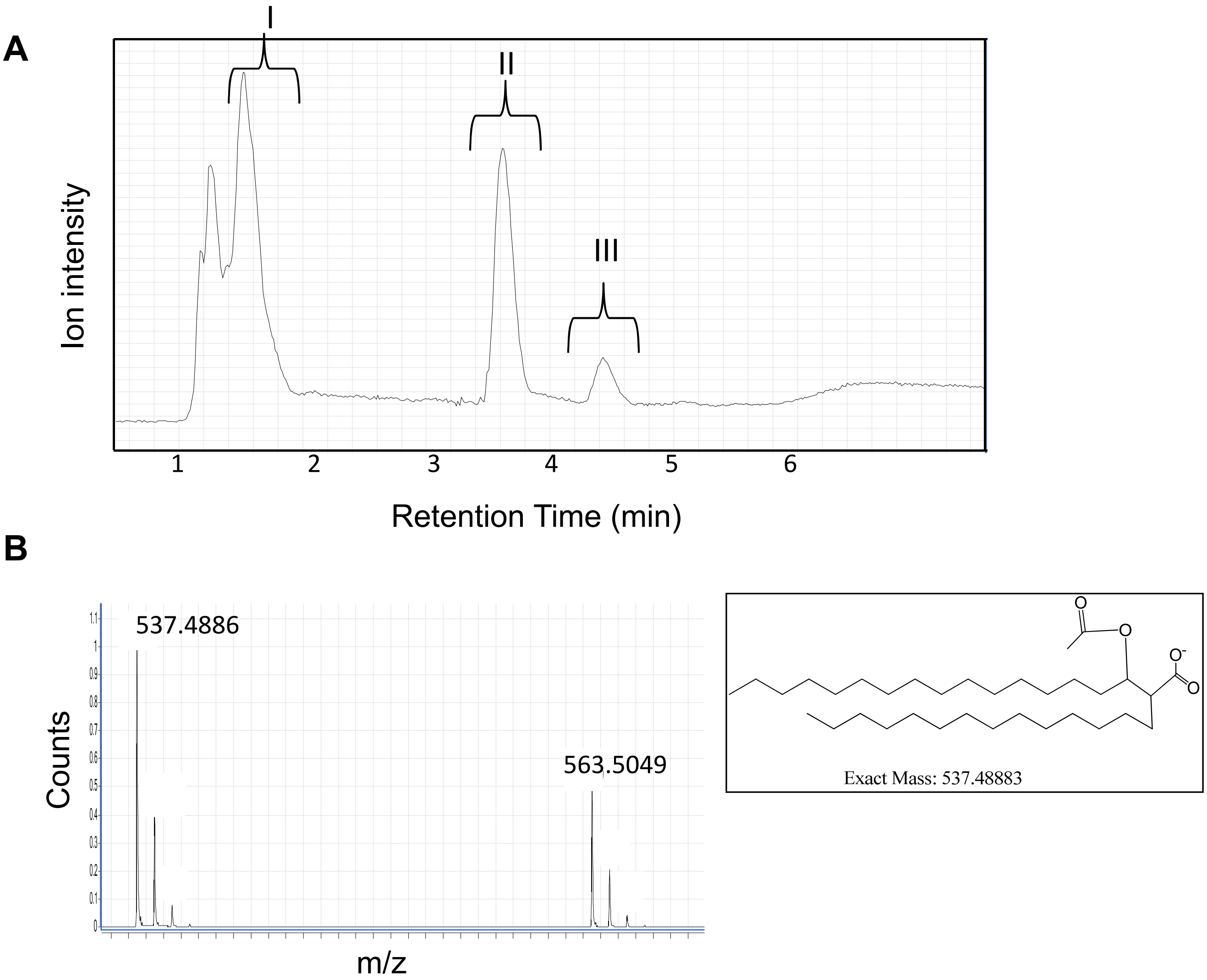

Fig. S4. (A) LC/MS chromatogram of Lipid X. Component I which barely stuck to the reversed phase LC column was not further analyzed and may or may not be part of the Lipid X TLC spot. Component III is like component II except it is based on a C-34 fully saturated corynomycolate. (B) The mass spectrum of component II consistent with acetylated C-32 fully saturated corynomycolate (m/z = 537.4886) whose structure is shown on the right) and monounsaturated C-34 corynomycolate (m/z = 563.5049).

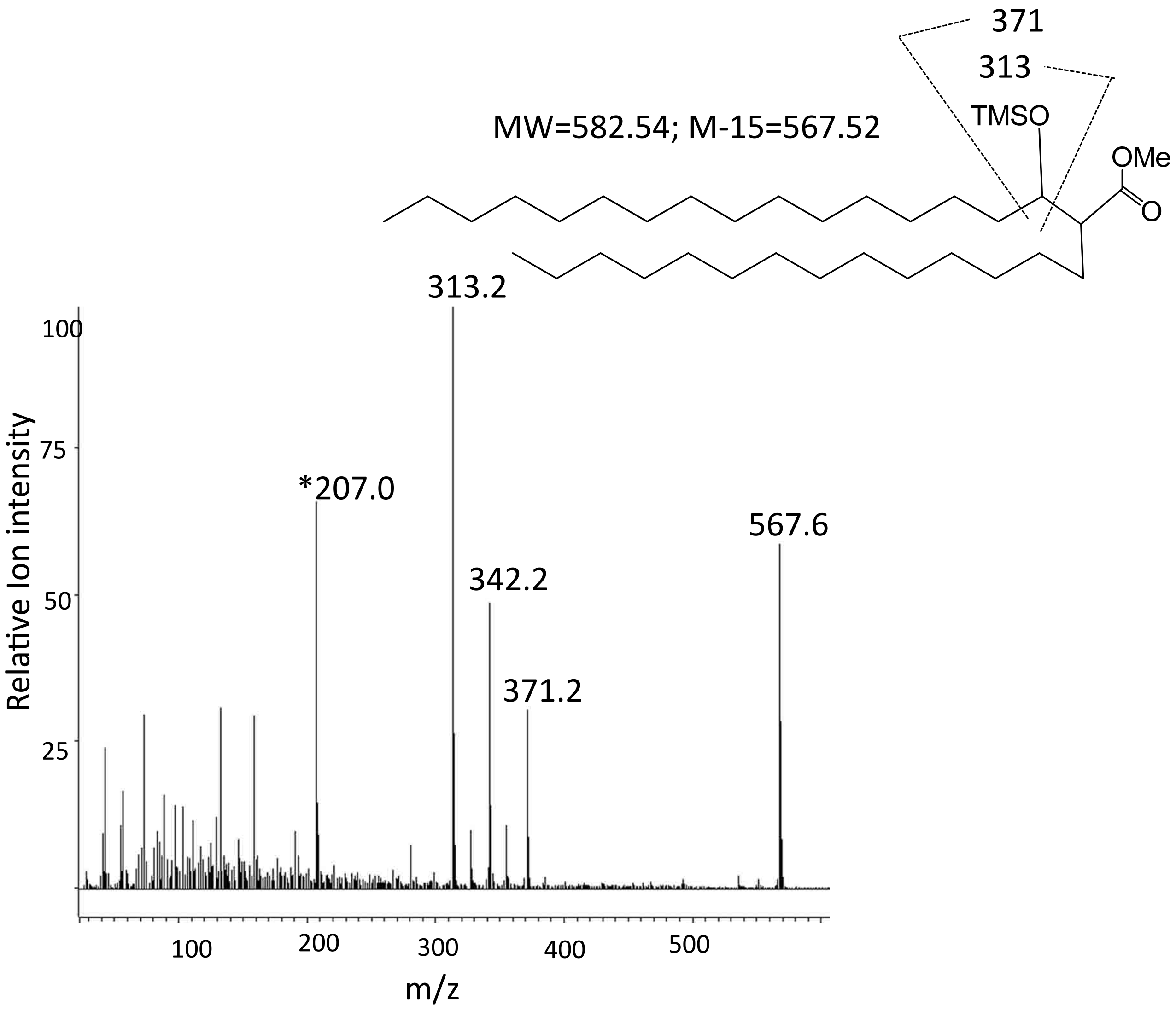

Fig. S5. The structure of the methyl ester trimethylsilyl ether of C-32 corynomycolate as determined by GC/MS after formation of the methyl ester and trimethylsilylation of the alcohol. The m/z 207 ion (asterisk) is from column bleed. M-15 is 567.6. The original LC/MS analysis also had a complex early eluting peak containing ions at m/z 485.3854, 489.3359, and 521.3622 and the GC/MS had many components in addition to the corynomycolates. These possible other components have not been investigated.
